# Advanced Oxidation Processes in Pharmaceutical Formulations: Photo-Fenton Degradation of Peptides and Proteins

**DOI:** 10.3390/ijms23158262

**Published:** 2022-07-27

**Authors:** Christian Schöneich

**Affiliations:** Simons Research Laboratories, Department of Pharmaceutical Chemistry, The University of Kansas, 2093 Constant Avenue, Lawrence, KS 66047, USA; schoneic@ku.edu

**Keywords:** photo-degradation, radicals, oxidation, Fenton reaction, charge transfer, peptide, protein

## Abstract

Formulations of therapeutic proteins are sensitive to photo-degradation by near UV and visible light. Mechanistically, especially the processes leading to protein modification under visible light exposure are not understood. Potentially, these processes may be triggered by a ligand to metal charge transfer in excipient-metal complexes. This article summarizes recent analytical and mechanistic work on such reactions under experimental conditions relevant to pharmaceutical formulations.

## 1. Introduction

Physical and chemical long-term stability are key factors controlling the development of efficacious and safe therapeutic protein formulations [1,2]. Potential problems associated with physical and chemical degradation of formulations include the formation of protein aggregates and particles, the loss of potency, and the generation of immunogenic material. Frequently, physical and chemical degradation processes are still treated as separate, unrelated events. However, there is strong evidence that physical stress can cause chemical modifications of proteins and/or excipients, and, vice versa, that chemical stress can lead to changes in the physical appearance of formulations. For example, the chemical modification of proteins can lead to partial unfolding, exposing hydrophobic domains available for protein aggregation [3]. In another example, mechanical shock can result in cavitation, promoting the formation of reactive oxygen species available for protein oxidation [4]. Hence, physical and chemical degradation in pharmaceutical formulations should always be explored with a focus on a potential cross-talk between these pathways.

Protein formulations are subject to various different chemical degradation reactions, including hydrolysis, isomerization, side-chain and backbone cleavage, and oxidation [2]. Here, details of oxidative degradation mechanisms are least understood, due to the fact that protein oxidation in formulations is not generally initiated by a known concentration of a well-characterized oxidant (equivalent, e.g., to a hydrolysis reaction catalyzed by a known and constant concentration of hydronium or hydroxide ion, controlled by pH). Instead, oxidants are frequently generated in situ during manufacturing and/or long-term storage of formulations or formulation components, or during sterilization processes. Information about the nature of oxidants generated in situ can often only be obtained through the detection of oxidation products characteristic of specific oxidants.

For the rational development of chemically stable protein formulations, the nature of reactive intermediates and the mechanisms by which they are generated during manufacturing and long-term storage must be understood. Recently, much emphasis has been placed on the characterization of photo-induced degradation processes, especially those triggered by exposure to visible light [5,6,7,8]. Generally, the individual amino acid building blocks of proteins contain no chromophores which absorb visible light. At best, the amino acids tryptophan (Trp), tyrosine (Tyr), histidine (His), and cystine display negligible absorbances of UV A light. However, visible and UV A light represent the predominant components of light to which protein formulations would be exposed during manufacturing, storage, and/or administration [5]. For example, indoor lighting (with window-filtered daylight) in a clinical setting was estimated to provide typical light intensities of 400–10,000 lux visible light and 17 Wm^−2^ UVA light [9]. Therefore, a 1 h intravenous infusion in a clinical setting would be exposed to a 400–10,000 lux h visible light dose and a 17 Whm^−2^ UV A light dose. The latter corresponds to 8.5% of the minimal UV light exposure (200 Whm^−2^) recommended for photo-stability testing by the current ICH Q1B guidelines [9].

Evidence has been provided that formulations of therapeutic proteins degrade during exposure to UV and visible light [5,6,7,8,10], as well as to ambient light [5], which is characterized as visible light with a small UV quotient [5]. Based on the known light absorption properties of individual amino acids, specifically the photo-degradation processes triggered by the exposure to visible or ambient light, cannot be rationalized by the photochemistry of individual amino acids. The rather low light intensities (see above) also exclude two-photon processes [11]. Photo-chemically active chromophores may be present as a result of impurities [12,13], protein post-translational modifications [11,14,15], cation-π complexes of aromatic amino acids [16,17,18], or charge transfer complexes, similar to those characterized for Trp and Tyr, for example in the presence of high salt concentrations [19,20,21,22].

As an alternative, we recently started to evaluate the potential role of near UV and visible light-induced photo-degradation reactions driven by ligand-to-metal charge transfer (LMCT) processes of pharmaceutical excipient-metal complexes, specifically, complexes of iron [23,24,25]. These reactions are part of a larger class of processes, generally referred to as advanced oxidation processes (AOPs) [26,27]. Currently, various AOPs are developed with a focus on water decontamination, including the removal of pharmaceuticals. Here, these processes are optimized with respect to reaction yields, reaction times, and economic factors. In pharmaceutical formulations, AOPs will not operate under conditions optimized for maximal yield. However, the fact that they may occur at all can put pharmaceutical formulations at risk to fail due to chemical instability problems. This account will summarize our recent mechanistic investigations designed to understand potential LMCT-dependent photo-degradation reactions in pharmaceutical formulations. This information may assist in the development of chemically stable protein formulations.

## 2. The Photo-Induced Oxidation of Model Peptides in Iron-Containing Citrate Buffer

Reactions (1)–(10) display a general sequence of processes for the generation of reactive oxygen species via photo-induced LMCT in a carboxylate-iron complex in an aqueous solution [28,29].

[Fe^3+^(RCO_2_^−^)_n_]^(3−n)+^ + hν → [Fe^2+^(RCO_2_^−^)_n−1_]^(2−(n−1))+^ + RCO_2_^•^ (1)

RCO_2_^•^ → R^•^ + CO_2_ (2)

[Fe^2+^(RCO_2_^−^)_n−1_]^(2−(n−1))+^ + O_2_ → [Fe^2+^(RCO_2_^−^)_n−1_]^(3−(n−1))+^ +O_2_^•−^ (3)

O_2_^•−^ + H^+^⇌HO_2_^•^ (4)

O_2_^•−^ + H^+^ + HO_2_^•^ → H_2_O_2_ + O_2_ (5)

Fe^2+^ + 2H^+^ + O_2_^•−^ → Fe^3+^ + H_2_O_2_ (6)

Fe^2+^ + H_2_O_2_ → Fe^3+^ + HO^−^ + HO^•^ (7)

R^•^ + O_2_ → ROO^•^ (8)

Fe^2+^ + ROO^•^ + H^+^ → Fe^3+^ + ROOH (9)

Fe^2+^ + ROOH → Fe^3+^ + HO^−^ + RO^•^ (10)

In this sequence, Reaction (7) is a simplified representation of the Fenton reaction: this process alone consists of several steps, including the formation of a Fe^2+^/H_2_O_2_ complex, followed by decomposition into hydroxyl radicals (HO^•^) or hypervalent iron-oxo species [30].

When air-saturated aqueous solutions containing 10 mM citrate, pH 6.0, and 50 µM Fe^3+^ were exposed over 120 min to a total light dose of 7.7 Whm^−2^ near UV light (Rayonet RPR 3500 lamps; light emission between 305–416 nm; λ_max_ = 350 nm), we detected the formation of H_2_O_2_ [23]. The yields of H_2_O_2_ increased over ca. 10 min to a peak concentration of ca. 35 µM, subsequently decomposing over the next 10 min. A gradual decrease in the Fe^3+^ concentrations to 10 and 5 µM resulted in an overall increase in the detectable H_2_O_2_ yields, and a delayed formation and decomposition. A further decrease in the Fe^3+^ concentrations to 1.0 and 0.5 µM resulted in a gradual increase in H_2_O_2_ yields over the entire 120 min photo-irradiation. These observations are consistent with an iron-dependent formation of H_2_O_2_ via Reactions (1)–(6), and an iron-dependent decomposition of H_2_O_2_ via Reaction (7).

From a pharmaceutical perspective, several points are important. First, the applied light dose of 7.7 Whm^−2^ near UV light amounts to < 4% of the UV light dose recommended for photo-stability studies by the ICH Q1B guidelines [9]. The light dose of 7.7 Whm^−2^ is in the range of the UV light dose a therapeutic protein may encounter during manufacturing (ca. 1.5–3.0 Whm^−2^) [5] and is below the light dose of a 1 h exposure to indoor lighting combined with window-filtered daylight in a clinical setting (see above) [9]. Second, iron concentrations of 1–9 µM have been quantified in formulations of therapeutic antibodies [31], i.e., iron concentrations in the range covered by the experiment described above. Third, several marketed formulations of therapeutic proteins contain (e.g., Acetris, Benlysta, Rituxan) (see respective package inserts) or originally contained (Humira) citrate buffer (current formulations of Humira are citrate-free [32]).

**Scheme 1:**
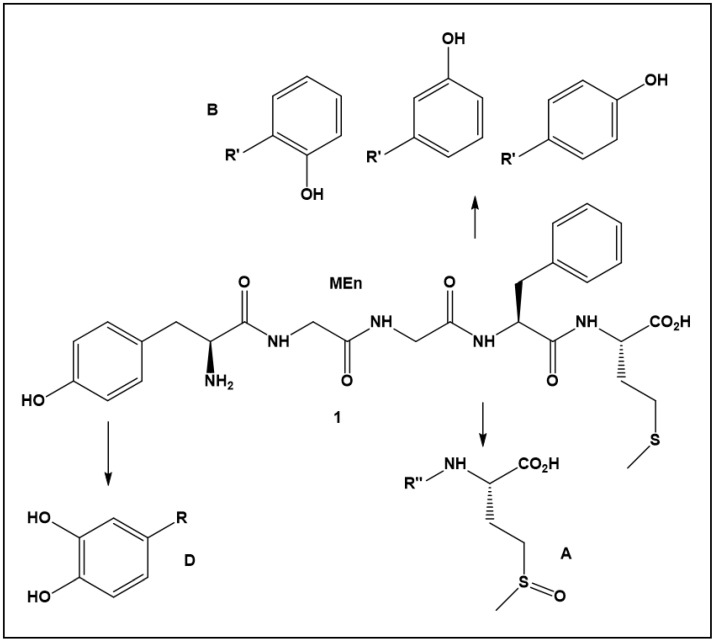
MEn and some oxidative degradation products **A**, **B** and **D** which were generated upon near UV light exposure (25 Whm^−2^) of 1 mM peptide in 10 mM citrate, pH 6.0, containing 0.5–50 µM. No structure for product **C** is given as a structure could not be unambiguously assigned (see text). For the degradation products, only the modified side chains are shown, whereas the remainder of the peptide structures is represented by substituents R, R′, and R″.

When air-saturated aqueous solutions containing 1 mM methionine (Met) enkephalin (MEn), 10 mM citrate, pH 6.0, and 5–50 µM Fe^3+^ were photo-irradiated with near UV light (25.2 Whm^−2^) several oxidation products of MEn were detected by HPLC-MS/MS analysis [23]. Here, MEn was added to the buffer as a model target peptide containing several oxidation-sensitive amino acids. The structure of MEn **1**, together with some of the detected oxidation products (**A**, **B,** and **D**) is shown in Scheme 1 (for product **C**, see below). The displayed oxidation products are expected based on the photo-chemical generation of H_2_O_2_ or ROOH (Reactions (5), (6) and (10)), i.e., Met sulfoxide (product **A**), and based on the formation of HO^•^ (Reaction (7)), i.e., the hydroxylation products dihydroxyphenylalanine (3-hydroxytyrosine; product **D**) and three regioisomers of hydroxyphenylalanine (products **B**). All these products were also detected upon photo-irradiation with visible light. In addition to these products, our HPLC-MS/MS analysis revealed several products originating from the N-terminal Tyr residue, namely products in which the Tyr residue was modified by +28, +56, +100, and +114 Da [23]. No unambiguous structural details are currently available for these products. In the original paper we referred to the Tyr (+28 Da) product as product **C**, and a series of analytical and mechanistic studies were performed in order to assign a structure and a formation mechanism to this product. Some of these studies will be described in more detail in the following as they illustrate that even in rather simple formulations containing a model peptide, buffer, and Fe^3+^, light exposure can lead to a number of novel products originating from currently unknown reaction mechanisms. Ultimately, these studies led to the identification of an important radical intermediate, relevant for the general formation of ROS and transformations of proteins in pharmaceutical formulations.

Product **C** decomposed during purification for NMR analysis, preventing any structural analysis by this means. Experiments under either a ^16^O_2_ or an ^18^O_2_ atmosphere revealed that product **C** incorporated a single oxygen atom from atmospheric oxygen, i.e., we obtained Tyr (+28 Da) under a ^16^O_2_ atmosphere and Tyr (+30 Da) under an ^18^O_2_ atmosphere. Hence, we hypothesized that a reaction intermediate *en route* to product **C** was N-terminal dihydroxyphenylalanine. The latter could theoretically convert into product **C** by reaction with formaldehyde. However, the addition of formaldehyde to photo-irradiated solutions of MEn did neither reduce the yields of dihydroxyphenylalanine nor increase the yields of product **C**. We independently showed that near UV photo-irradiation of 10 mM citrate, pH 6.0, containing 50 µM Fe^3+^ generated formaldehyde, but for comparison, photo-irradiation of 10 mM oxalate, pH 6.0, containing 50 µM Fe^3+^ did not generate formaldehyde [23]. Yet, product **C** was generated by photo-irradiation of 1 mM MEn in either 10 mM citrate or 10 mM oxalate, pH 6.0, containing 50 µM Fe^3+^, further excluding formaldehyde in the formation of product **C**. Moreover, the formation of product **C** did not require Tyr to be N-terminal, as an analogous Tyr modification was also detected for the model peptide Arg-Tyr-Leu-Pro-Thr (RYLPT).

The formation of product **C** during photo-irradiation of MEn in citrate and oxalate buffer suggested a common precursor generated in both buffers. Based on the photo-chemical generation of the C_2_O_4_^•−^ radical and carbon dioxide radical anion, ^•^CO_2_^−^, from Fe^3+^/oxalate [33] we hypothesized that light exposure of Fe^3+^/citrate could lead to the production of ^•^CO_2_^−^. Evidence for the photo-induced formation of ^•^CO_2_^−^ in citrate buffer will be presented in the following. 

## 3. Photo-Induced Formation and Reactions of ^•^CO_2_^−^ in Iron-Containing Citrate Buffer

**Scheme 2:**
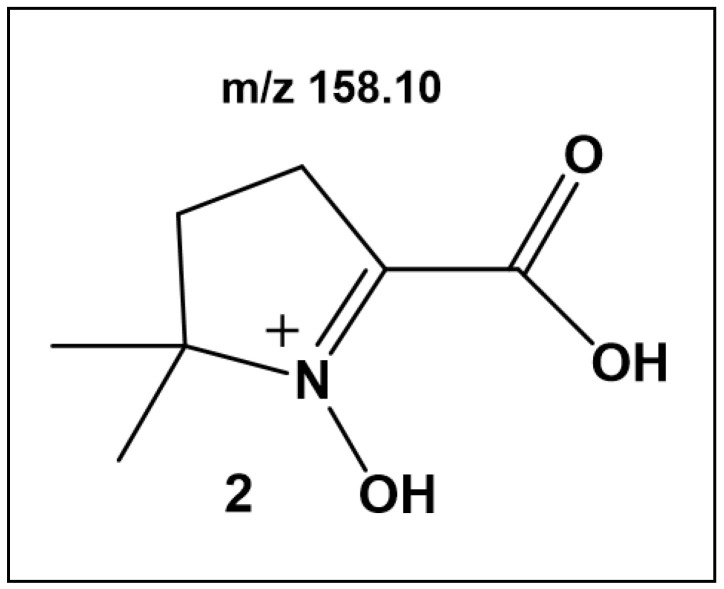
Structure of nitrone **2** with *m*/*z* 158.10, detected by HPLC-MS analysis.

When solutions containing 10 mM oxalate or citrate, but not succinate or acetate, pH 6.0, 50 µM Fe^3+^, and 50 mM 5,5-dimethyl-1-pyrroline N-oxide (DMPO) were exposed to 6.8 Whm^−2^ near UV light, HPLC-MS analysis revealed the formation of nitrone **2** (with *m*/*z* 158.10) [23] as displayed in Scheme 2.

**Scheme 3:**
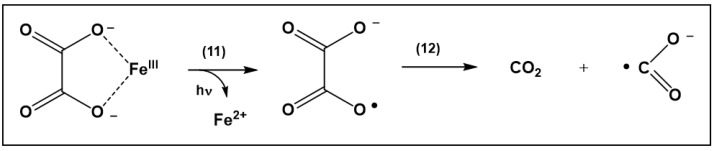
Photo-induced generation of ^•^CO_2_^−^ from oxalate.

Nitrone **2** is generated through the addition of ^•^CO_2_^−^ to DMPO, followed by disproportionation, which would also yield the corresponding hydroxylamine product (structure not shown). More recent studies (Zhang et al., unpublished results) have confirmed this reaction sequence for both oxalate and citrate buffer, demonstrating the initial formation of a nitroxide by electron spin resonance (ESR) spectroscopy, followed by conversion of the nitroxide into nitrone and hydroxylamine (detected by HPLC-MS/MS analysis). The generation of ^•^CO_2_^−^ from oxalate can be rationalized by Reactions (11) and (12), as displayed in Scheme 3.

Mechanistically, the generation of ^•^CO_2_^−^ from citrate is more complex. Initially, we had proposed that ^•^CO_2_^−^ could be generated via alkoxyl radical formation at either C_2_ or C_4_ of citrate, followed by the β-cleavage of ^•^CO_2_^−^ [23]. However, our more recent studies with ^13^C-labeled citrate point to LMCT from the central hydroxyl group of citrates, generating an intermediary alkoxyl radical at C_3_, followed by β-cleavage of ^•^CO_2_^−^ from the central carboxylate group (unpublished results).

The photo-induced formation of ^•^CO_2_^−^ expands the number of processes by which ROS can be generated in citrate buffer. Here, ^•^CO_2_^−^ reacts efficiently with Fe^3+^ and O_2_ via Reactions (13) [34,35] and (14) [35,36,37,38].

^•^CO_2_^−^ + Fe^3+^ → CO_2_ + Fe^2+^ (13)

^•^CO_2_^−^ + O_2_ → CO_2_ + O_2_^•−^ (14)

Moreover, ^•^CO_2_^−^ could be involved in the formation of product **C** described above. Considering, that dihydroxyphenylalanine may be an important intermediate in the formation of product **C**, we evaluated whether semiquinone radicals of the latter may be generated under light exposure of MEn in citrate buffer. Upon inclusion of DMPO, we detected a DMPO-adduct to dihydroxyphenylalanine, suggesting indeed the intermediary generation of dihydroxyphenylalanine semiquinone radicals. Therefore, it is possible that the mechanism for the formation of product **C** includes a reaction of a dihydroxyphenylalanine semiquinone radical with ^•^CO_2_^−^. The formation of product **C** would then require chemical transformation(s) of such ^•^CO_2_^−^/dihydroxyphenylalanine semiquinone radical adduct, but any further conclusions must await the results of additional analytical and mechanistic experiments. Nevertheless, the generation of ^•^CO_2_^−^ during near UV (and visible) light exposure of citrate buffer is an important detail relevant to the stability of pharmaceutical formulations.

The photo-chemical generation of ^•^CO_2_^−^ in citrate buffer enables additional fundamental processes of protein degradation. Radiation chemical studies [39,40,41] have long demonstrated the efficient reduction of small molecular weight and protein disulfides by ^•^CO_2_^−^ (Reactions (15) and (16)), where frequently ^•^CO_2_^−^ is generated by the exposure of aqueous solutions of formate to oxidizing radicals produced by ionizing radiation.

^•^CO_2_^−^ + R-S-S-R → CO_2_ + [R-S-S-R]^•−^ (15)

[R-S-S-R]^•−^ + H^+^ → RSH + RS^•^ (16)

Consistent with the formation of ^•^CO_2_^−^, the photo-irradiation of experimental citrate formulations, containing Fe^3+^, polysorbate 80, and disulfide-containing peptides (glutathione disulfide, octreotide) or a protein (insulin), resulted in the formation of free thiols, detected via derivatization with ThioGlo1 [25]. Moreover, the intermediary generation of thiyl radicals was documented by cis/trans-isomerization of unsaturated fatty acids in the surfactant polysorbate 80 [25].

## 4. Photo-Induced Oxidation in the Absence of Added Iron

From a pharmaceutical perspective, it was important to examine whether LMCT-dependent photo-degradation would occur in the absence of added iron, i.e., whether basal iron impurities in citrate buffer would be sufficient for product formation. For this, we quantified basal iron levels in citrate buffers made from various lots of different suppliers. When 10 mM citrate buffer was prepared from five different lots, the concentrations of Fe^3+^ varied between ca. 0.02 and 0.22 µM [23]. Importantly, upon photo-irradiation of 1 mM MEn in these citrate buffers, pH 6.0, with 25.2 Whm^−2^ near UV light, the yields of Met sulfoxide correlated strongly with the measured basal Fe^3+^ concentrations. Under these experimental conditions of rather low Fe^3+^ concentrations, Met sulfoxide was the only significant product [23]. 

## 5. Photo-Induced Oxidation Processes in the Presence of Amino Acids

**Scheme 4:**
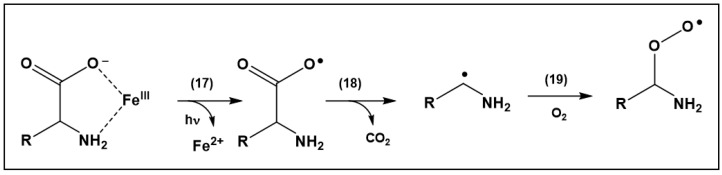
Proposed mechanism for the photo-induced generation of peroxyl radicals from amino acid/Fe^3+^ complexes.

When citrate buffer was replaced by acetate or succinate, photo-irradiation generated significantly lower yields of H_2_O_2_ (where yields were higher in succinate as compared to acetate buffer) [23]. The addition of MEn to acetate buffer led to low yields of Met sulfoxide (product **A**). However, for both citrate and acetate buffer, the addition of common amino acid excipients, specifically lysine (Lys) and histidine (His), significantly increased the yields of Met sulfoxide from MEn [24]. These increased yields of Met sulfoxide were not caused by an increased formation of H_2_O_2_. Instead, we proposed that LMCT processes in amino acid/Fe^3+^ complexes caused the formation of amino acid-derived peroxyl radicals, which directly oxidized Met to Met sulfoxide [24]. A potential mechanism for peroxyl radical formation is displayed in Scheme 4 (Reactions (17)–(19)), a representative for any peroxyl radical generated via the addition of O_2_ to an intermediary carbon-centered radical.

Noteworthy, the addition of free Met to the formulations efficiently protected against the oxidation of the Met residue in MEn. However, specifically in citrate buffer, reaction products from free Met, such as methional, reacted with the N-terminal Tyr residue of MEn, generating a condensation product [24].

## 6. Conclusions and Outlook

There is a growing interest in the mechanisms, which lead to the photo-induced degradation of therapeutic proteins specifically under exposure to visible and ambient light. For example, Sreedhara et al. [5] and Kaiser et al. [7] showed that ambient and visible light exposure led to the modification of several monoclonal antibodies. However, mechanistic analysis of the underlying photo-processes is currently rather difficult as different antibodies and antibody concentrations in different formulations were compared. These formulations contained either one or more buffers, and sometimes amino acids. Kaiser et al. [7] only mention buffers in their formulations but not whether additional amino acids or surfactants were present. Our model studies on advanced oxidation processes described here show that amino acids such as Lys, His, or arginine (Arg) can promote the oxidation of Met residues in target peptides [24], suggesting that a similar effect may also operate with proteins. In the absence of added amino acids, therapeutic proteins can contain peptide sequences/domains which complex metals, enabling site-directed LMCT-dependent photo-degradation processes within specific protein domains even in the absence of added amino acids. Future studies on ambient and visible light-induced protein degradation should include the possibilities of such reactions in search of the reaction mechanisms for light-induced protein degradation.

## Data Availability

Not applicable.
